# Oxidative stress and inflammation in hemodialysis: a comparison of patients with or without advanced nonalcoholic fatty liver disease (NAFLD)

**DOI:** 10.1080/0886022X.2025.2455523

**Published:** 2025-01-22

**Authors:** Vanja Kalacun, Robert Ekart, Sebastjan Bevc, Pavel Skok, Radovan Hojs, Nina Vodošek Hojs

**Affiliations:** a Department of Gastroenterology, Clinic for Internal Medicine, University Medical Centre Maribor, Maribor, Slovenia; b Department of Dialysis, Clinic for Internal Medicine, University Medical Centre Maribor, Maribor, Slovenia; c Faculty of Medicine, University of Maribor, Maribor, Slovenia; d Department of Nephrology, Clinic for Internal Medicine, University Medical Centre Maribor, Maribor, Slovenia

**Keywords:** Nonalcoholic fatty liver disease, hemodialysis, elastography, oxidative stress, inflammation

## Abstract

Nonalcoholic fatty liver disease (NAFLD) and chronic kidney disease are global public health issues associated with high morbidity and mortality. Both diseases are also interlinked. Little is known about the meaning of NAFLD in hemodialysis (HD) patients. Therefore, the aim of our study was to investigate the difference in oxidative stress and inflammation in HD patients with or without advanced NAFLD. Seventy-seven HD patients were included (65.14 ± 12.34 years, 59.2% male) and divided according to abdominal ultrasound and two-dimensional shear wave elastography (2D-SWE) measurements into two groups: 1) no NAFLD or no advanced NAFLD (2D-SWE <9 kPa) and 2) advanced NAFLD (2D-SWE ≥9 kPa). Medical history data and blood results were collected. HD patients with advanced NAFLD had significantly higher levels of 8-hydroxy-2’-deoxyguanosine (8-OHdG; *p* = 0.025), tumor necrosis factor-alpha (TNF-α; *p* = 0.023), and intercellular adhesion molecule 1 (ICAM-1; *p* = 0.015) in comparison to HD patients without advanced NAFLD. Interleukin 6 (IL-6) was higher in the advanced NAFLD group, but the difference was of borderline significance (*p* = 0.054). There was no significant difference in high-sensitivity C-reactive protein (hs-CRP), and vascular cell adhesion molecule 1 (VCAM-1) between groups. In binary logistic regression analysis, advanced NAFLD was significantly associated with 8-OHdG and ICAM-1. In conclusion, higher oxidative stress and inflammation levels are present in HD patients with advanced NAFLD.

## Introduction

Nonalcoholic fatty liver disease (NAFLD) is defined histologically as hepatic steatosis in more than 5% of hepatocytes, in the absence of other causes of chronic liver disease (excessive alcohol consumption, viral, autoimmune hepatitis, etc.). NAFLD is divided into nonalcoholic fatty liver (NAFL) and nonalcoholic steatohepatitis (NASH). NAFL represents hepatic steatosis without significant inflammation, while in NASH significant inflammation and hepatocellular injury is present. Therefore, NASH represents a more severe and serious form of NAFLD. Patients with NASH can progress to even more advanced disease, including fibrosis, cirrhosis, and hepatocellular carcinoma [1,2]. Patients with NASH or advanced liver fibrosis have a higher risk of liver-related, cardiovascular, and overall mortality [2–6]. NAFLD increases the risk of extra-hepatic diseases such as type 2 diabetes mellitus, dyslipidemia, metabolic syndrome, hypertension, cardiovascular or cerebrovascular diseases, and chronic kidney disease (CKD) [6]. In the global population, the prevalence of NAFLD is approximately 30%, whereas for NASH, it is 5% [7,8].

For the diagnosis of NAFLD, hepatic steatosis needs to be demonstrated by imaging or biopsy. Different radiologic methods can detect NAFLD, but none of them can differentiate between different histologic subtypes of NAFLD. Although liver biopsy remains the gold standard to diagnose NAFLD, grade steatosis, necrosis, inflammation, and to assess fibrosis, it is rarely performed because of its invasive nature. With the increasing prevalence of NAFLD, and the growing clinical and research need to assess NAFLD severity, other noninvasive and more available methods are necessary. Since the stage of liver fibrosis is one of the most important predictors of outcome, different noninvasive methods to assess liver stiffness/fibrosis have been developed, including elastographic methods involving ultrasonography and magnetic resonance imaging. The four main methods are vibration-controlled transient elastography, point shear wave elastography (pSWE), two-dimensional shear wave elastography (2D-SWE), and magnetic resonance elastography. All of them have excellent and similar diagnostic accuracy in detecting liver fibrosis in NAFLD [9–11]. Vibration-controlled transient elastography is the first tool with which liver stiffness was measured and studied. It is ultrasound-guided, but without direct image guidance, and a specific device Fibroscan^®^ (Echosens, Paris, France) is needed [12,13]. pSWE and 2D-SWE can both be done as an add-on during liver ultrasonography (using the same probe), can be performed with real-time imaging, so liver capsule, large vessels, bile ducts, and focal masses can be avoided, multiple regions of the liver can be assessed, they are low-cost, easily accessible, repeatable and convenient to use [13]. In 2020, the Society of Radiologists in Ultrasound Liver Elastography proposed a vendor-neutral ‘rule of four’ (5, 9, 13, 17 kPa) for pSWE and 2D-SWE techniques for viral etiologies and NAFLD [14]. Liver stiffness of 5 kPa or less has a high probability of being normal; liver stiffness less than 9 kPa, in the absence of other known clinical signs, rules out compensated advanced chronic liver disease; values between 9 kPa and 13 kPa are suggestive of compensated advanced chronic liver disease; and values greater than 13 kPa are highly suggestive of compensated advanced chronic liver disease.

Various studies have shown that NAFLD is associated with a higher risk of CKD [5,15]. A large meta-analysis also indicates that NAFLD is significantly associated with increased long-term risk of incident CKD stage ≥3 [16]. CKD prevalence in NAFLD has been estimated to be approximately 20–55% [17]. However, also ultrasonographically documented fatty liver disease was shown to be an independent risk factor for nonfatal cardiovascular events in hemodialysis (HD) patients [18]. Oxidative stress, dyslipidemia, and inflammation are tightly and independently related to NAFLD [15]. Oxidative stress activates the inflammatory cascade derived from visceral adipose tissue and adipokines and cytokines lead to impaired insulin sensitivity [19]. An important effect of oxidative stress is also impaired endothelial function which is a well-known predictor of future cardiovascular events [20]. Oxidative stress and inflammation are also suggested to be the key factors in the pathogenesis and progression of CKD in NAFLD patients [15,21]. Less is known about the meaning of NAFLD and its association with oxidative stress and inflammation in HD patients. This is important because of the development of new treatment strategies and possible improvement of HD patients’ outcomes. Therefore, the aim of our study was to investigate oxidative stress and inflammation in HD patients with advanced NAFLD, defined by 2D-SWE.

## Methods

### Participants

We invited all 117 HD patients from our department to participate in the study. The inclusion criteria were: signed informed consent, age more than 18 years, HD treatment of at least 6 months before inclusion. Twenty patients refused to participate; all others gave signed consent. From screening till the beginning of the study seven patients died and two were transplanted. The exclusion criteria were: active malignant disease (4 patients), pregnancy (none), active infection during the examinations (none), myocardial infarction or stroke in the last 3 months before inclusion (5 patients), known liver disease of other etiology, except NAFLD (2 patients). The remaining 77 HD patients were additionally screened for unhealthy alcohol use with the AUDIT (Alcohol Use Disorders Identification Test) and CAGE (Cut, Annoyed, Guilty, and Eye) questionnaire. No test showed problematic drinking of alcohol in the included patients. All our HD patients are regularly tested for hepatitis B and C infection, and all included patients were negative.

### Study design

At the time of inclusion, medical history data and standard laboratory blood results were collected, including high-sensitivity C-reactive protein (hs-CRP). hs-CRP was measured with a routine procedure using Siemens Healthineers Dimension Vista^®^ System (Siemens Healthcare Diagnostics Products GmbH, Marburg, Germany, Cat.No. K7’046). Additionally, blood was withdrawn for tumor necrosis factor-alpha (TNF-α), interleukin 6 (IL-6), vascular cell adhesion molecule 1 (VCAM-1), intercellular adhesion molecule 1 (ICAM-1) and 8-hydroxy-2′-deoxyguanosine (8-OHdG). All samples were analyzed in batch mode from frozen samples (-85 °C). The measurement of 8-OHdG content in plasma was performed using an ELISA kit (R&D Systems, Inc., Minneapolis, USA, Cat. no. 4380-096K) according to the manufacturer’s procedure. The same procedure was used for the determination of ICAM-1 (R&D systems, Inc., Minneapolis, USA, Cat.no. DCD540) and VCAM-1 (R&D systems, Inc., Minneapolis, USA, Cat.no. DVC00). The concentration of IL-6 and TNF-α in serum samples was determined on an IMMULITE^®^ 1000 system (Siemens Healthcare Diagnostics Products GmbH, Marburg, Germany) using two-site solid-phase chemiluminescence immunometric assays (Cat. No. LKSP1 and LKNF1, respectively).

Abdominal ultrasound and 2D-SWE measurements of the liver were performed by one experienced gastroenterologist with a Philips ultrasound system (Epiq 5, Philips Healthcare, Bothell, WA, USA). Both examinations were performed on a non-dialysis day during the short interdialytic interval. Patients fasted for at least 6 h before the examination. All measurements were performed according to the guidelines from the Society of Radiologists in Ultrasound [13,14]. We did at least ten independent measurements in each patient. Based on the result of the abdominal ultrasound we defined patients as having or not having NAFLD. 2D-SWE helped us divide patients with NAFLD into those with or without advanced NAFLD.

The study was in accordance with the Declaration of Helsinki and The Code of Ethical Conduct of the University of Maribor [22]. It was approved by the national ethics committee (0120-358/2022/3, approved October 4, 2022), and all patients gave written informed consent.

### Statistical analysis

Data are presented as absolute numbers and percentages for categorical data, as mean ± standard deviation for normally distributed numerical data, and as median and interquartile ranges for non-normally distributed data. For further analysis data of TNF-α, IL-6, VCAM-1, ICAM-1, and 8-OHdG were naturally log-transformed because of non-normal distribution.

Patients were divided into two groups according to ultrasound and liver stiffness cutoff values assessed by 2D-SWE recommended by society guidelines: (1) no NAFLD or no advanced NAFLD (2D-SWE <9 kPa) and (2) advanced NAFLD (2D-SWE ≥9 kPa) [14]. Data of both groups were compared by the independent sample t-test or Mann-Whitney test. Additionally, we performed binary logistic regression analysis, advanced NAFLD was the dependent variable and as covariates we used log-transformed data of TNF-α, IL-6, VCAM-1, ICAM-1, and 8-OHdG.

All data were analyzed with SPSS version 27.0 (IBM SPSS, Chicago, IL, USA). A *p*-value <0.05 was considered statistically significant.

## Results

**Figure 1.**
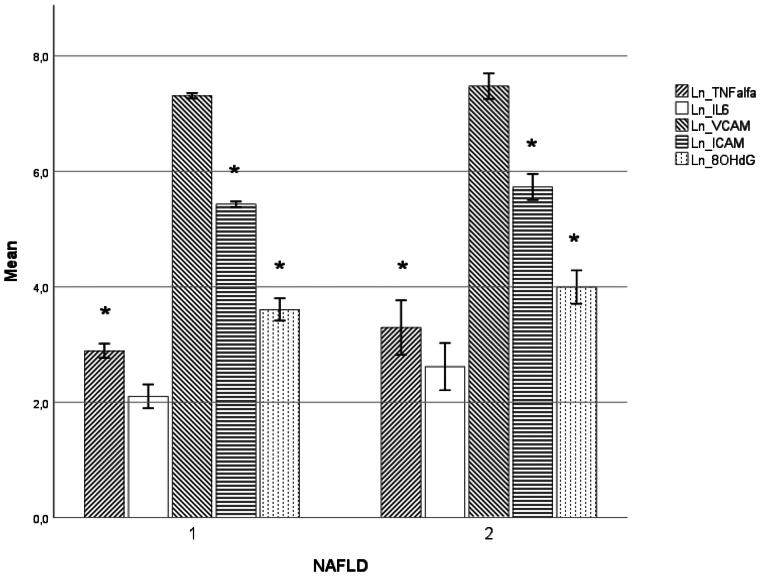
Naturally log-transformed data of TNF-α, IL-6, VCAM-1, ICAM-1, and 8-OHdG in no NAFLD or no advanced NAFLD (NAFLD = 1) and in advanced NAFLD (NAFLD = 2). NAFLD 1 = No NAFLD or no advanced NAFLD (2D-SWE <9 kPa) NAFLD 2 = Advanced NAFLD (2D-SWE ≥9 kPa) *= statistically significant.

We included 77 HD patients, causes of end-stage renal disease are presented in Table 1. 46 (59.2%) were male, and the average age was 65.14 ± 12.34 years. 97.4% had arterial hypertension and 32.5% had diabetes mellitus type 1 or 2. 26 (33.8%) HD patients had ultrasonographically visible hepatic steatosis. The average 2D-SWE value of all patients was 6.37 ± 2.69 kPa. All our HD patients were dialyzed with online hemodiafiltration and synthetic membranes, three times weekly for 4 h. We divided our patients according to ultrasound and 2D-SWE results into two groups, data of them are presented in Table 2. Both groups of patients had normal values of liver tests, except for elevated gamma-glutamyl transferase (GGT) in the advanced NAFLD group. Groups of patients did not differ significantly in age, sex, presence of diabetes, cardiovascular diseases, body mass index (BMI), dialysis vintage or adequacy (Kt/V), hemoglobin, aspartate aminotransferase (AST), alanine aminotransferase (ALT), alkaline phosphatase (ALP), serum albumin, total cholesterol, low-density lipoprotein (LDL) cholesterol, high-density lipoprotein (HDL) cholesterol, neither triglycerides. Serum ferritin and serum cholinesterase (CHE) were significantly lower in the advanced NAFLD group, while GGT, total, and conjugated bilirubin were significantly higher in this group of patients. hs-CRP was lower in the advanced NAFLD group, but the difference was not significant. IL-6 was higher in the advanced NAFLD group but of borderline significance (*p* = 0.054). Patients with advanced NAFLD had significantly higher TNF-α, ICAM-1, and 8-OHdG (Figure 1). VCAM-1 did not differ significantly between groups. In binary logistic regression analysis, advanced NAFLD was significantly associated with 8-OHdG (*p* = 0.040) and ICAM-1 (*p* = 0.016).

**Table 1.** Causes of end-stage renal disease (ESRD) in our 77 hemodialysis patients included in the study.

Causes of ESRD	Number
Hypertensive nephropathy	24
Diabetic kidney disease	17
Obstructive nephropathy/Chronic pyelonephritis	10
Adult polycystic kidney disease	9
Glomerulonephritis (biopsy proven)	6
Other	5
Systemic vasculitis	2
Alport syndrome	1
Lowe syndrome	1
Light chain deposition disease	1
Unknown	6

**Table 2.** Data of 77 hemodialysis patients divided according to the result of ultrasound and two-dimensional shear wave elastography (2D-SWE) of the liver into: no nonalcoholic fatty liver disease (NAFLD) or no advanced NAFLD (<9 kPa) and advanced NAFLD (≥9 kPa).

	No NAFLD or no advanced NAFLD (2D-SWE <9 kPa)	Advanced NAFLD (2D-SWE ≥9 kPa)	*p*-value
Number of patients (%)	66 (85.7%)	11 (14.3%)	/
Age (years)	65.45 ± 12.48	63.27 ± 11.84	0.590
Male sex (%)	59.1	63.6	0.777
Diabetes mellitus type 1 or 2 (%)	31.8	36.4	0.767
Cardiovascular diseases (%)	54.5	72.7	0.262
BMI (kg/m^2^)	27.42 ± 4.99	26.39 ± 7.20	0.555
Dialysis vintage (days)	2,056.64 ± 2321.88	2,252.00 ± 2804.97	0.803
Kt/V	1.24 ± 0.23	1.25 ± 0.24	0.965
Hb (g/L)	112.56 ± 13.93	114.91 ± 13.58	0.605
Ferritin (µg/L)	305.73 ± 139.81	216.91 ± 94.03	**0.046**
AST (µkat/L)	0.18 ± 0.07	0.26 ± 0.23	0.273
ALT (µkat/L)	0.29 ± 0.10	0.36 ± 0.22	0.326
GGT (µkat/L)	0.51 ± 0.40	1.28 ± 1.08	**0.040**
ALP (µkat/L)	1.42 ± 0.57	1.53 ± 0.56	0.561
Conjugated bilirubin (µmol/L)	2.45 ± 0.64	3.64 ± 1.36	**0.016**
Total bilirubin (µmol/L)	7.55 ± 2.28	10.55 ± 3.48	**0.018**
CHE (µkat/L)	192.78 ± 51.80	143.09 ± 59.18	**0.005**
Serum albumin (g/L)	33.69 ± 3.72	33.82 ± 2.56	0.914
Cholesterol (mmol/L)	4.36 ± 1.18	3.76 ± 0.81	0.110
LDL cholesterol (mmol/L)	2.36 ± 0.94	1.95 ± 0.76	0.169
HDL cholesterol (mmol/L)	1.28 ± 0.45	1.37 ± 0.80	0.730
Triglycerides (mmol/L)	1.62 ± 1.06	1.51 ± 1.00	0.745
hs-CRP (mg/L)	8.07 ± 12.68	6.65 ± 6.55	0.719
TNF-α (pg/mL)	17.4; 14.3–21.9	19.9; 16.2–45.7	**0.023**
IL-6 (pg/mL)	8.3; 4.6–12.3	14.0; 9.6–18.8	0.054
VCAM-1 (ng/mL)	1,468; 1,414–1,558	1,468;1,468–1,858	0.134
ICAM-1 (ng/mL)	224.0; 200.0–253.50	247.0; 237.0–383.0	**0.015**
8-OHdG (ng/mL)	38.8; 23.2–62.6	47.8; 39.3–72.8	**0.025**

Legend: BMI: body mass index; Kt/V: dialysis adequacy; Hb: hemoglobin*;* AST: aspartate aminotransferase*;* ALT: alanine aminotransferase; GGT: gamma-glutamyl transferase; ALP: alkaline phosphatase; CHE: serum cholinesterase; LDL: low-density lipoprotein; HDL: high-density lipoprotein; hs-CRP: high sensitivity C-reactive protein; TNF-α: tumor necrosis factor-alpha; IL-6: interleukin 6; VCAM-1: vascular cell adhesion molecule 1; ICAM-1: intercellular adhesion molecule 1; 8-OHdG: 8-hydroxy-2’-deoxyguanosine.

Values of *p* < 0.05 were considered statistically significant and are bolded.

## Discussion

We found that HD patients with advanced NAFLD had significantly higher levels of oxidative stress and inflammation in comparison to HD patients without NAFLD or without advanced NAFLD. Additionally, oxidative stress and inflammation (ICAM-1) were significant predictors of advanced NAFLD.

We measured oxidative stress in our patients with 8-OHdG, an oxidative product of DNA and a sensitive biomarker of oxidative stress, which can reflect extremely low levels of oxidative DNA damage [23]. DNA damage causes cellular damage and is associated with various chronic and degenerative diseases [24].

8-OHdG is a surrogate marker of oxidant-induced DNA damage in dialysis patients [25,26]. Tarng et al. found the greatest 8-OHdG level in leukocyte DNA in HD patients, followed by non-dialysis CKD patients and healthy controls [26]. Interestingly, Domenici et al. found that vitamin E supplementation significantly reduced 8-OHdG in HD patients [27]. Similarly, Tarng et al. found that vitamin C supplementation did the same in HD patients [28]. However, in both studies, the effect of vitamin supplementation on clinical outcomes was not studied. Oxidative stress is associated with endothelial dysfunction, so it is not surprising that 8-OHdG in HD patients is related to higher cardiovascular and all-cause mortality [20,29–31]. Dai et al. have shown that 8-OHdG is also associated with increased mortality risk in CKD patients, independent of inflammation [23]. Our results suggest that patients with advanced NAFLD have higher levels of oxidative stress and inflammation, whether they also have higher cardiovascular risk should be proven in further prospective studies.

8-OHdG is also of relevance in NAFLD. Seki et al. immunohistochemically studied hepatic expression of 8-OHdG in healthy human liver and NAFLD [32]. No 8-OHdG expression was found in normal liver and only in a few with fatty liver, but in 64.7% with NASH. The 8-OHdG index significantly correlated with the grade of necro-inflammation [32]. Irie et al. examined the expression of 8-OHdG in liver biopsy specimens and serum of patients with simple fatty liver and NASH [33]. The percentage of hepatocytes positive for 8-OHdG expression and serum 8-OHdG levels were significantly higher in patients with NASH than in simple fatty liver. Also, Jiang et al. found the highest serum levels of 8-OHdG in liver biopsy-proven NASH in comparison to NAFLD and healthy controls [34]. These studies confirm the important difference between simple fatty liver and NASH. 8-OHdG is also associated with hepatocarcinogenesis in NAFLD. Kakehashi et al. investigated cellular and molecular alterations in the liver, and hepatocellular carcinomas in NASH model 60-week-old Tsumura, Suzuki, Obese Diabetic (TSOD) mice [35]. Among other discoveries, they found a marked accumulation of 8-OHdG in the liver and altered foci in these mice. This was associated with a rise in inflammation (TNF-α, IL-6), fibrosis markers, and cell proliferation. Therefore, they postulated that the formation of 8-OHdG in the DNA of TSOD mice liver cells could be an early event in type 2 diabetes mellitus/NASH-associated hepatocarcinogenesis. In another study, higher liver 8-OHdG in liver biopsy-proven NAFLD patients was related to higher serum α-fetoprotein, hepatocellular ballooning, stage of fibrosis, and higher accumulation of methylated tumor suppressor genes (loss of function of these genes) [36]. The last might be associated with hepatocarcinogenesis in NAFLD. However, oxidative stress could also be a risk for carcinogenesis through methylation-independent pathways. In one study antioxidant therapy with glutathione reduced the serum 8-OHdG in NASH patients [37]. It is not known if oxidative stress is associated with malignancies in HD patients.

8-OHdG in our HD patients with advanced NAFLD was significantly higher than in patients without advanced NAFLD. This is similar to the previously mentioned studies done in NAFLD patients without CKD. To the best of our knowledge, only one study was published on oxidative stress in HD patients with NAFLD. They included 71 nondiabetic HD patients, and 19 (26.8%) of them had NAFLD [38]. In comparison to our study, NAFLD was defined only by ultrasound and no additional techniques. After measuring blood levels of different oxidative stress markers (thiobarbituric acid reactive substances, free thiols, superoxide dismutase activities, and glutathione peroxidase activity), they found that only glutathione peroxidase activity (antioxidant) was higher in patients with NAFLD. They assumed this reflected a heightened response to the oxidative stress associated with NAFLD. No 8-OHdG was measured in this study.

In our study, HD patients with advanced NAFLD had significantly higher levels of TNF-α, and ICAM-1. IL-6 was higher in the advanced NAFLD group, but the difference was of borderline significance. There was no significant difference between the groups in VCAM-1. It is not unexpected that patients with higher oxidative stress have also higher levels of inflammation since oxidative stress induces it. Interestingly, hs-CRP was lower in the advanced NAFLD group, but without significant difference between groups. Studies regarding inflammatory markers in HD patients with NAFLD are scarce. As far as we know, only data on CRP in this group of patients is published. Mikolasevic et al. performed a study on 72 HD patients and 50 sex- and age-matched controls [39]. They used a different technique to ours to define NAFLD in HD patients, namely vibration-controlled transient elastography, 38 (52.8%) HD patients had NAFLD. Comparison of CRP levels showed significant differences between all groups and in group-to-group comparisons. The highest CRP was present in NAFLD patients (16.7 ± 6.8 mg/L), followed by non-NAFLD patients (4.6 ± 0.9 mg/L) and controls (1.2 ± 0.9 mg/L). In another study by Mikolasevic et al. they also used vibration-controlled transient elastography to define NAFLD [40]. 53 (56.4%) out of 94 HD patients had NAFLD. They found a positive correlation between the severity of liver steatosis and hs-CRP. Mikolasevic et al. also performed a study on elderly HD patients with NAFLD, defined by vibration-controlled transient elastography [41]. Out of 76 patients, 44 (57.9%) had NAFLD. They found a positive correlation between NAFLD and hs-CRP. Furthermore, the presence of NAFLD and high hs-CRP were strong predictors of poor outcome. Stolic et al. also studied elderly HD patients with NAFLD [42]. NAFLD was defined only by ultrasound, 37 (51%) of 72 patients had NAFLD. The median CRP values of the non-NAFLD and NAFLD groups were 4.1 and 6 mg/L, respectively, without significant differences between groups. Lai et al. also used ultrasound to define NAFLD in 278 HD patients [18]. Fatty liver was identified in 78 (28%) patients. Interestingly, hs-CRP was lower in the fatty liver group, but the difference was not statistically significant. In univariate analysis, hs-CRP showed only a weak positive correlation with nonfatal cardiovascular events, in multivariate analysis the correlation was insignificant. Behairy et al. explored the presence of NAFLD in CKD stage 3-5 and HD patients with vibration-controlled transient elastography [43]. Of 50 HD patients, 29 (58%) had NAFLD and they were divided into two groups according to the grade of liver steatosis (grade 1, grade 2 + 3). Patients with grade 2 + 3 steatosis had significantly higher CRP levels than grade 1 or non-NAFLD patients. CRP levels did not differ significantly between non-NAFLD and stage 1 steatosis.

Interestingly, both groups of our patients had normal liver tests (AST, ALT, ALP, bilirubin, CHE) except for GGT, which was slightly elevated and significantly higher in the advanced NAFLD group. The advanced NAFLD group had significantly higher total and conjugated bilirubin, while CHE was significantly lower in this group. The latter could be a sign of reduced synthesis due to liver dysfunction in the advanced NAFLD group. However, serum albumins which are also a marker of synthetic function of the liver, were at the lower end but still within the normal range in both groups. Both groups did not differ significantly in serum albumin levels.

Limitations of our study are that we included only Caucasian patients, the number of patients is relatively small, and the study was performed in a single center. It was a cross-sectional study, and no cause-effect can be determined. We did not perform a liver biopsy to determine the scale of inflammation and fibrosis in NAFLD patients. However, in addition to basic imaging, such as ultrasound, we also used 2D-SWE to assess liver fibrosis. Other strengths of our study are the inclusion of stable HD patients, the inclusion of patients’ medical histories, laboratory parameters, and different markers of inflammation.

In conclusion, higher oxidative stress and inflammation are present in HD patients with advanced NAFLD. Inflammation and oxidative stress are also predictors of advanced NAFLD. Since 2D-SWE of the liver is unharmful and relatively simple to perform, it could help us in recognizing HD patients with advanced NAFLD and consequently a higher risk of all-cause and cardiovascular morbidity and mortality. Further studies are needed on the effect of advanced NAFLD on the morbidity and mortality of these patients. It is also unknown if anti-oxidative stress therapy is useful in HD patients with advanced NAFLD in improving their outcomes. Therefore, further prospective studies are needed to answer these questions and to clarify the meaning of our findings.

## Data Availability

The datasets generated and analyzed during the current study are available from the corresponding author on reasonable request.
